# Loss of SMARCAD1 Mitigates Tauopathy

**DOI:** 10.1111/acel.70543

**Published:** 2026-05-27

**Authors:** Vaishnavi S. Jadhav, Rebecca L. Kow, Asia D. Beale, Misa Baum, Pamela J. McMillan, Caitlin S. Latimer, Nicole F. Liachko, Brian C. Kraemer

**Affiliations:** ^1^ Division of Gerontology and Geriatric Medicine, Department of Medicine University of Washington Seattle Washington USA; ^2^ Geriatrics Research Education and Clinical Center Veterans Affairs Puget Sound Health Care System Seattle Washington USA; ^3^ Department of Psychiatry and Behavioral Sciences University of Washington Seattle Washington USA; ^4^ Department of Laboratory Medicine and Pathology University of Washington Seattle Washington USA; ^5^ Mental Illness Research, Education, and Clinical Center Veterans Affairs Puget Sound Health Care System Seattle Washington USA; ^6^ Department of Genome Sciences University of Washington Seattle Washington USA

**Keywords:** Alzheimer's disease, *C. elegans*, epigenetic, HEK‐tau, *SMARCAD1*, *smrd‐1*, tau, tauopathy

## Abstract

Tauopathies are neurodegenerative diseases characterized by the accumulation of misfolded tau protein and include Alzheimer's disease (AD) and related dementia disorders. Identifying new strategies to treat tauopathy remains an important gap in the field. Using forward and reverse genetic approaches in *C. elegans* , we identified *smrd‐1*, the *C. elegans* homolog of *SMARCAD1*, as a potent modifier of tauopathy phenotypes in a transgenic model of tauopathy. Loss of *smrd‐1* function rescues tauopathy‐associated neuronal dysfunction and neurodegeneration in *C. elegans* models of tauopathy. Loss or reduction of *smrd‐1/SMARCAD1* decreases phosphorylated and total tau protein levels by reducing tau mRNA transcripts in *C. elegans* and mammalian HEK‐tau cells. Loss of *smrd‐1* rescues tau‐driven abnormal H3K9me3 chromatin methylation. Immunohistochemistry in human postmortem AD brain tissue showed SMARCAD1 depletion in a subset of cases that also exhibit depletion of MSUT2. Loss of *smrd‐1/SMARCAD1* rescues tau‐mediated neurodegeneration via a tau mRNA lowering mechanism accompanied by changes in chromatin conformation.

## Graphical abstract

Using genetics approaches in a *C. elegans* transgenic model of tauopathy, we uncover a role for the homolog of human SMARCAD1, a regulator of chromatin dynamics.

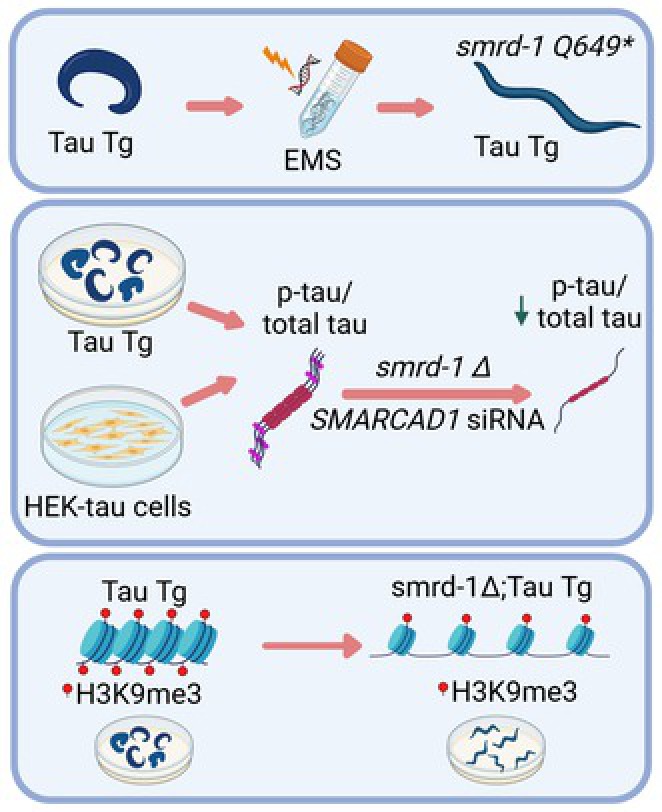

**Abbreviations**
AD: Alzheimer's disease
ADNC: Alzheimer's disease neuropathic change
ATP: Adenosine triphosphate
CRISPR: Clustered Regularly Interspaced Short Palindromic Repeats
DEG: Differentially expressed genes
FTLD: Fronto‐Temporal Lobar Degeneration
GABA: Gamma‐aminobutyric acid
GO: Gene Ontology
H3K9me3: Trimethylation of Lys9 of histone H3
HDAC6: Histone Deacetylase‐6
HEK293: Human embryonic kidney cells clone 293
MAPT: Microtubule Associated Protein Tau
N2:
*C. elegans* Bristol strain
NGM: Nematode Growth Media Agar plates
PS19: MAPT P301S mouse model
Tg: Transgenic
UDP: Uridine diphosphate
WT: Wildtype

## 1 Introduction

Tauopathies encompass a broad spectrum of neurodegenerative disorders characterized by accumulation of fibrillar polymers of the microtubule‐associated protein tau (MAPT) (Götz et al. 2019). Deposition of misfolded protein tau leads to neurodegeneration resulting in a range of symptoms including cognitive decline, behavioral abnormalities, motor dysfunction, dementia, and premature death. There are currently over 20 distinct tauopathy disorders identified, including frontotemporal dementia and Alzheimer's disease (Lane‐Donovan and Boxer 2024). Tau pathology strongly correlates with cognitive dysfunction (Bejanin et al. 2017; Brier et al. 2016; Nelson et al. 2012). Currently, no effective treatments for tauopathies have been approved. Therefore, identifying modifiers of pathological tau and potential drug targets to mitigate tauopathies remains a critical area of research.

We have previously developed a *C. elegans* model of tauopathy. In this model, neuronal expression of human tau leads to accumulation of hyperphosphorylated and insoluble tau, accompanied by progressive neuronal dysfunction and neuron loss (Kraemer et al. 2003). Forward and reverse genetic screens in this model have previously identified novel conserved tau suppressors including *suppressor of tau pathology‐1* (*sut‐1*), *suppressor of tau pathology‐2* (*sut‐2/MSUT2*), *suppressor of tau pathology‐6* (*sut‐6/NIPP1*), *X‐box binding protein 1* (*xbp‐1s/XBP‐1s*), and *speckle‐type POZ protein* (*spop‐1/SPOP*) (Eck et al. 2023; Guthrie et al. 2009; Kow et al. 2023; Kraemer and Schellenberg 2007; Waldherr et al. 2019). Loss of *MSUT2* reduced tau pathology in murine models, and in human postmortem tissue lower MSUT2 levels correlated with early onset of AD (Wheeler et al. 2019). Using a similar approach in this study, we have identified a new tau suppressor *smrd‐1/M03C11.8* (*SMARCAD1* human homolog). SWI/SNF‐related, Matrix‐associated Actin‐dependent Regulator of Chromatin, subfamily A, containing DEAD/H box 1 (SMARCAD1), an ATP‐dependent chromatin remodeler, is conserved from yeast to humans, and its function is critical for DNA replication and homologous recombination following dsDNA breaks repair (Chakraborty et al. 2018; Lo et al. 2021). SMARCAD1 also functions in the propagation and organization of epigenetic patterns as well as in heterochromatin maintenance and inheritance (Rowbotham et al. 2011; Taneja et al. 2017). SMARCAD1 is recruited to newly synthesized DNA and shown to facilitate histone deacetylation, histone H3K9 trimethylation (H3K9me3), and efficient HP1 recruitment through a mechanism coupled to ATP hydrolysis (Rowbotham et al. 2011).

While SMARCAD1 knockdown in human cancer cell lines has been shown to impair metastasis and invasion (Al Kubaisy et al. 2016), its role in other diseases, such as neurodegeneration, remains largely unknown. Here in this study, we investigate SMARCAD1 as a novel tau suppressor in *C. elegans* and human cell line models of tauopathy.

## 2 Methods

### 2.1 *C. elegans* Strains

*C. elegans* strains used are listed in Table S1. The *C. elegans* alleles *bk2196* and *bk4016* were generated through chemical mutagenesis as previously described and subsequently outcrossed 3× (Kow et al. 2023). The *smrd‐1 null* strains CK3122 and CK3123 were generated using the CRISPR‐Cas9 genome editing technology using ALT‐R reagents (Integrated DNA Technologies) including recombinant Cas9 protein and synthetic CRISPR guide RNAs as described (Dokshin et al. 2018; Kow et al. 2021; Paix et al. 2015). The crRNA guide sequences (TTTTTAGGGGAAATTAATGG and ATATTTTTCGGTGCTTTTCG) targeting exons 1–6 that result in deletion of the Helicase/ATP‐binding region of the *smrd‐1* were used. All other strains were obtained from the *C. elegans* Genetics Center. All strains were maintained at 20°C on NGM plates seeded with OP50 *Escherichia coli* (Brenner 2003). Genotypes were confirmed by PCR and sequencing or restriction digests for all strains with non‐obvious phenotypes.

### 2.2 Forward Genetic Mutagenesis Screen

Mutagenesis was performed using N‐ethyl‐N‐nitrosourea (ENU) as described (De Stasio and Dorman 2001). Briefly, tau transgenic worms were exposed to 0.6 mM ENU in M9 for 4 h with gentle rocking followed by selection of F2 progeny with the desired mutant phenotype of restored motility in the presence of the tau transgene (Kraemer and Schellenberg 2007). Mutants that bred true were then tested on an independent tau transgenic background. Suppressing mutants were backcrossed repeatedly to remove non‐causal mutations prior to whole‐genome sequencing (WGS) to identify tau suppressing mutations. Genome sequence analysis was conducted using the Lasergene genomics SeqMan NGen software package (DNASTAR).

### 2.3 Behavior in *C. elegans*

NGM plates of day 1 adult *C. elegans* were flooded with 1 mL of M9 buffer, and swimming worms were pipetted onto a 35 mm unseeded NGM plate. Approximately 30 s following the addition of M9 buffer, worms were recorded swimming for 1 min at 14 frames per second. These videos were captured and analyzed with WormLab 2020 (MBF Bioscience). The frequency of body bends, or turns, defined as a change in body angle of at least 20° from a straight line measured by the quarter points and midpoint of the worms, was quantified as a readout of locomotion. Worms tracked for less than 40 s were omitted from this analysis. At least three independent samples totaling approximately 80 worms per strain were counted for every comparison.

### 2.4 GABAergic Neuron Loss Assay

A blinded analysis of GABAergic inhibitory motor neuron loss was performed by using a *C. elegans* strain expressing GFP under the *unc‐25* promoter (P*unc*‐25::GFP). This reporter strain (CZ1200) was crossed into tau transgenic animals to allow GABAergic neuron visualization in vivo. Day 1 adult worms were immobilized on a 2% agarose pad with 0.01% sodium azide and imaged using a Leica DM6B fluorescence microscope. GABAergic neurons were counted while blinded to the genotype for each mounted worm. The number of intact neurons was scored.

### 2.5 HEK Tau Cells and siRNA Treatment

HEK 293/tau cells, HEK 293 cell lines stably expressing high levels of wild‐type human tau, as described previously, were maintained under standard culture conditions [Dulbecco's modified Eagle Medium, 10% heat inactivated fetal bovine serum, penicillin (100 U/mL), streptomycin (100 μg/mL) + Zeocin (100 μg/mL)] (Guthrie and Kraemer 2011). For RNAi, 1 E6 cells per 10 cm plate were plated 24 h before treatment in 10 mL full media. Per plate, 300 pmol SMARCAD1 13.6 siRNA (IDT) was diluted in 1 mL Opti‐MEM (Gibco) and mixed with 30 μL Lipofectamine RNAiMAX (Invitrogen) diluted in 1 mL Opti‐MEM and incubated 5 min at room temperature. Media was replaced 24 h after treatment and cells were harvested 96 h post‐treatment.

### 2.6 Protein Extraction and Immunoblotting

*C. elegans* were grown from hypochlorite‐purified eggs at 20°C for 3 days on 5XPEP plates until young adults. Worm lysates were prepared as described previously (Kow et al. 2023). Briefly, snap‐frozen worm pellets were thawed and then diluted with 2× SDS protein sample buffer, adding four times the volume (μL) of buffer to the weight of the pellet (mg). Samples were sonicated, centrifuged, and stored at −20°C. HEK293/tau cell lysates were prepared as described previously (Guthrie and Kraemer 2011). Briefly, HEK tau cell pellets were resuspended (2 μL lysis buffer/mg pellet) in high salt reassembly (RAB) buffer and sonicated at 70% amplitude for 8 s. 5X sample buffer was added to the lysate. Samples were loaded (10 μL) onto 4 to 15% precast criterion sodium dodecyl sulfate polyacrylamide gel electrophoresis gradient gels and transferred to PVDF (Bio‐Rad). The ladder is Precision Plus Protein Standards (Bio‐Rad). The primary antibodies used were rabbit monoclonal anti‐tau antibody (Dako) at 1:200,000, mouse anti‐tubulin antibody E7 (Developmental Studies Hybridoma Bank) at 1:5000, mouse anti‐pS202/T203 tau AT8 antibody (ThermoFisher) at 1:1000, mouse anti‐tau PHF‐1 antibody (Peter Davies) at 1:1000, rabbit anti‐SMARCAD1 antibody (Booster Bio), rabbit anti‐H3K9me3 (Millipore) at 1:2000. The secondary antibodies used were anti‐rabbit HRP (Fisher) at 1:10,000 and anti‐mouse HRP (Fisher) at 1:5000. ECL substrate was used to visualize the membrane (Bio‐Rad). Chemiluminescence signals were detected with the ChemiDoc‐It Imager (Analytik Jena US Inc.) and quantified with Fiji (45).

### 2.7 RNA Extraction and Quantitative PCR

RNA was extracted from L4 snap‐frozen *C. elegans* pellets using TRIzol Reagent (Thermo Fisher Scientific Inc.) per manufacturer's protocol. RNA was resuspended in 50 μL sterile water and concentration was assessed using NanoPhotometer NP80 spectrophotometer (Implen GmbH). DNA contamination was then removed from the RNA sample using the DNA‐free Kit (Thermo Fisher). For HEK 293/tau cells, RNA was extracted from frozen pellets using TRIzol reagent followed by column extraction using QIAGEN mini Rneasy as recommended by the manufacturer. cDNA and NoRT negative controls were synthesized using iScript Reverse Transcription Supermix for RT‐qPCR (Bio‐Rad Laboratories Inc.). We performed quantitative PCR using the iTaq Universal SYBR Green Supermix kit (Bio‐Rad) on the CFX Connect Real‐Time PCR Detection System (Bio‐Rad) to assess mRNA levels of human *MAPT* in relation to *act‐1* (Table S2). Each genotype was tested in three to four biological replicates and three technical replicates. MAPT mRNA levels were normalized to *rpl‐32* or *act‐1*, internal reference control genes and expressed as fold changes relative to controls, using the ΔΔCt method.

### 2.8 RNA Sequencing Library Prep, Quality Control, Sequencing

Isolated total RNA from L4 *C. elegans* was sent to NovoGene where library preparation and mRNA sequencing was performed using the NovaSeq platform (Illumina) to generate 150 bp paired end reads. FASTQC was performed to assess the quality of sequencing data. Illumina RNA sequencing reads were aligned to the Ensemble Caenorhabditis_elegansWBcel235.113 reference genome using STAR version 2.7.11b. Counting was performed with featureCounts. Read normalization and differential gene expression analyses were conducted with DESeq2, comparing *smrd‐1 null* to N2. Genes with a Benjamini‐Hochberg adjusted *p* value ≤ 0.05 were considered differentially expressed and were visualized using Volcano plot (using ggplot package in R) and heatmap (using pheatmap package in R).

### 2.9 GO Classification and Gene Enrichment Analyses

The Database for Annotation, Visualization and Integrated Discovery (DAVID) online tool was used to identify significantly enriched GO terms (*p* value < 0.05) in biological process, cellular component, and molecular function for DESeq and DEXSeq analyses (Huang et al. 2009; Sherman et al. 2022). Gene ontology using WormEnrichR was performed on significant genes with *p*‐adjusted < 0.05 using DAVID.

### 2.10 Immunohistochemistry in Human Post‐Mortem Tissue

Samples of post‐mortem brain tissue (middle temporal gyrus) were obtained from the University of Washington BioRepository and Integrated Neuropathology (BRaIN) Laboratory (Table S3). Informed consent for research brain donation was obtained according to protocols approved by the UW and KPWHRI Institutional Review Boards. Alzheimer's disease brain donors (*n* = 39) were chosen based upon the clinical diagnosis of dementia and neuropathologically confirmed Alzheimer's disease neuropathologic change (ADNC) sufficient to explain dementia (intermediate or high). Brain tissues used as controls for this study (*n* = 18) were derived from age‐matched cognitively normal research participants with neuropathologically confirmed low levels of ADNC. Fixation of donor brains occurred by immersion in 10% neutral buffered formalin for at least 2 weeks. The medial temporal gyrus was processed and embedded in paraffin and sectioned at 5‐μm thickness according to routine protocols for neuropathological analysis as described below.

### 2.11 Immunohistochemical Evaluation

The dementia cases in this cohort were immunostained for MSUT2, a marker of nuclear speckles, and classified as either Dementia MSUT2 positive (*n* = 17) or Dementia MSUT2 depleted (*n* = 22). Control cases (*n* = 18) were all MSUT2 positive. The Dementia MSUT2 depleted cases exhibited diminished MSUT2 immunoreactivity compared to control brain tissue, both in the number of MSUT2 positive neurons and in the intensity of the staining and were characterized as “MSUT2 negative.” In contrast, the Dementia MSUT2 positive cases exhibited similar amounts of MSUT2 immunoreactivity compared to control brain and were considered as “MSUT2 positive.” This scoring is made by blinded observers. Immunohistochemistry for SMARCAD1 was performed on tissue from this same cohort. Brain sections were deparaffinized, rehydrated through ethanols and autoclaved (SMARCAD1) or microwaved (AT180) in citrate buffer for antigen retrieval. Sections were treated for endogenous peroxidases, blocked in 5% milk, incubated with anti‐SMARCAD1 polyclonal antibody (Origene TA332959; 1:500) or AT180 (Thermo Scientific MN1040; 1:250) overnight at 4°C, followed by biotinylated rabbit or mouse secondary antibody. Sections were incubated with an avidin‐biotin complex (Vector, Vectastain Elite ABC kit) and the reaction product was visualized with 0.05% diaminobenzidine (DAB)/0.01% hydrogen peroxide. Digital images were obtained using a Leica DM6 microscope with a DFC 7000 digital camera (Leica Microsystems) and imported into Adobe Photoshop.

### 2.12 Quantitative Analysis of Immunohistochemistry

HALO digital image software (Indica Labs) was used to quantify SMARCAD1 and AT180 immunoreactivity in human brain. Brain sections were manually annotated around the regions of interest (cortical layers 3–6); average staining intensity was determined to allow quantification without contribution of background staining, and a common threshold was then applied to all sections for that assay. Data represent the area of positive immunoreactivity within the region of interest divided by the total annotated area (% SMARCAD1 positive tissue). Data are displayed as the mean ± SEM. A two‐tailed Student's *t*‐test was used to assess differences in immunoreactivity between experimental groups.

### 2.13 Statistical Analysis

Statistical analysis was performed using GraphPad Prism version 9 for Windows (GraphPad Software). The tests used were Student's *t*‐test, Mann–Whitney test, one‐way analysis of variance (ANOVA) test followed by Tukey's post hoc test, and Kruskal–Wallis test followed by Dunn's post hoc test. Statistical significance is demarcated in figures as *p < 0.05, **p < 0.01, ***p < 0.001, and ****p < 0.0001.

## 3 Results

### 3.1 Loss of *smrd‐1* Function Rescues Tauopathy Mediated Behavioral Deficits

**FIGURE 1:**
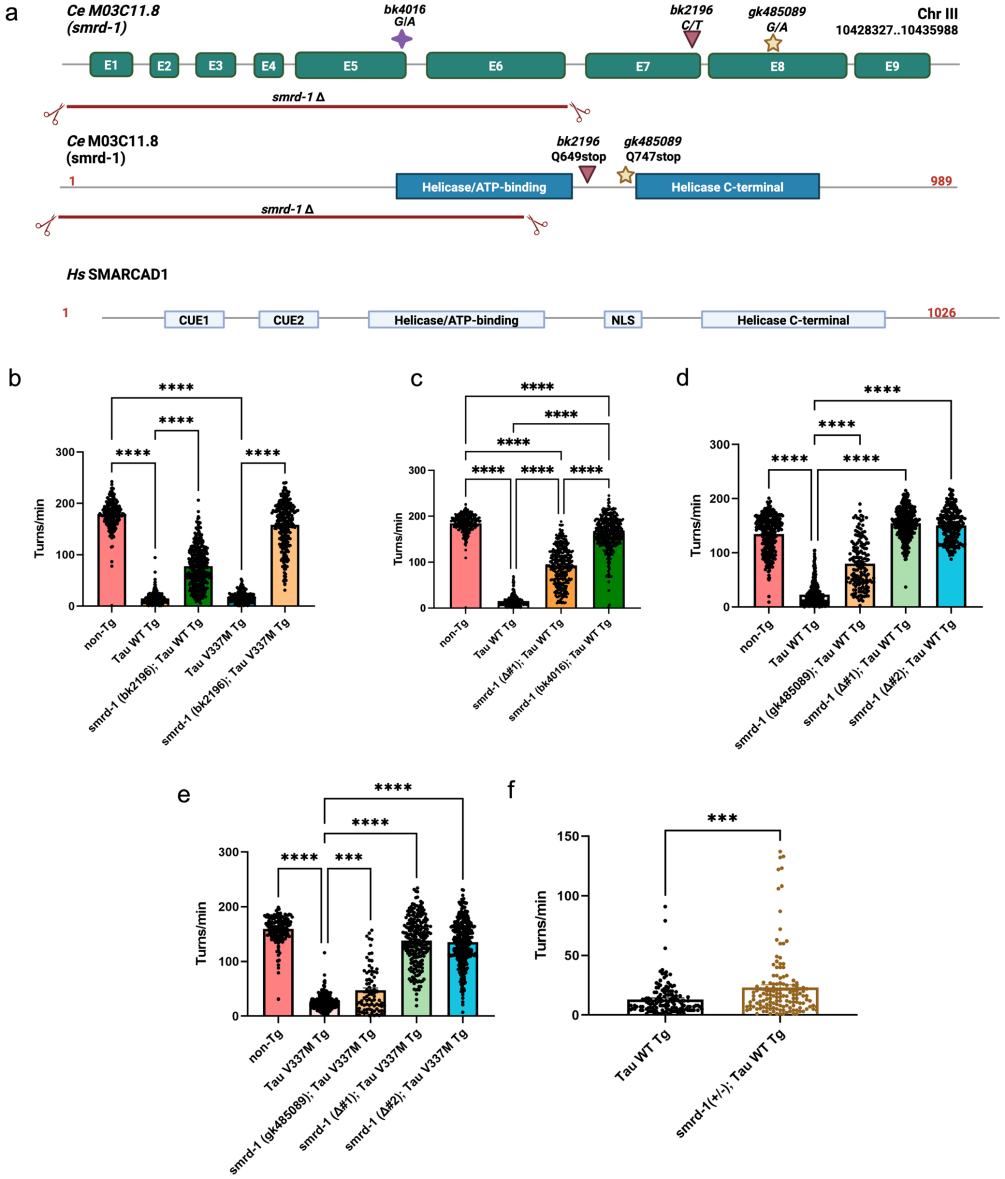
Loss of *smrd‐1* selectively attenuates tauopathy associated behavior deficits. (a) Illustration of *smrd‐1* alleles used in the study. (b) *smrd‐1* (*bk4016*), significantly rescued motor deficits Tau WT Tg animals. (c) *smrd‐1* (*bk2196*) rescued tau mediated motor deficits in Tau WT and *V337M* Tg animals. (d, e) *smrd‐1* (*gk485089*), *smrd‐1* (*Δ#1*), and *smrd‐1* (*Δ#2*) rescued tauopathy associated swimming defects in both animals with (d) Tau WT Tg background and in (e) Tau *V337M* Tg background. (f) Heterozygous *smrd‐1* (*Δ#1*) rescues tau associated motor deficits in Tau WT Tg animals. *N* > 80 per strain. Data presented as mean ± SEM frequency of body bends (turns/min) measured over the course of a minute of swimming in day 1 adults and analyzed by One way ANOVA with Tukey's post hoc test. ****p < 0.0001, ***p < 0.001.

Using a forward genetic screen for identifying genes that suppress the tau‐induced Unc phenotype in our previously described tau transgenic *C. elegans* model (Kraemer et al. 2003; Kraemer and Schellenberg 2007), we identified two strong tau suppressor mutations in *smrd‐1* (*M03C11.8*), whose human homolog is SWI/SNF‐related, Matrix‐associated Actin‐dependent Regulator of Chromatin, subfamily A, containing DEAD/H box 1 (*SMARCAD1*). The first *smrd‐1* suppressor mutant identified introduces a premature stop codon (bk2196‐Q649*). The second *smrd‐1* suppressor mutant (*bk4016*) harbors a point mutation at the splice junction of intron 5 predicted to disrupt exon 6 inclusion and cause a frameshift and premature stop after exon 5. To confirm the suppressors, we tested an additional independent premature stop *smrd‐1* allele (gk485089‐Q747*) and generated CRISPR‐Cas9 mediated deletion of *smrd‐1* exons 1–6 (*smrd‐1 Δ#1*, *Δ#2*) (Figure 1a) (Kow et al. 2021; Thompson et al. 2013). To assess neuronal function in tau transgenic strains, we use a swimming assay that provides a sensitive readout of GABAergic and cholinergic motor neuron activities. Tau Tg animals exhibit significant impairment in their coordinated swimming behavior (Kraemer et al. 2003). Using this assay, we observed *smrd‐1* loss of function results in a significant rescue of Tau Tg swimming deficits. We found similar improvements with the loss of function *smrd‐1* alleles identified in the screen (*bk2196*, Figure 1b, *bk4016* Figure 1c) and in the additional *smrd‐1* alleles *gk485089* and *smrd‐1 Δ#1*, *Δ#2* (Figure 1d,e), in both Tau WT and FTLD‐tau mutant Tau *V337M* background. There were no significant alterations in behavioral phenotypes of loss of function alleles in the absence of tau (Figure S1a,b).

To examine whether a partial loss of *smrd‐1* would protect against tau swimming deficits, we tested animals heterozygous for the *smrd‐1* loss of function, *smrd‐1 Δ#1* strain *CK2935*. These animals demonstrated modest yet significant rescue of Tau WT (Figure 1f and Figure S1c). In order to test whether rescue in locomotor deficits by the loss of functional *smrd‐1* is specific to tauopathy, we crossed the *smrd‐1* ∆#1 deletion allele with animals expressing familial ALS mutant TDP‐43 M337V and did not observe any suppression in swimming defects (Figure S1d). However, unlike the *aex‐3* promoter used to drive tau expression in Tau WT, the TDP‐43 M337V transgene tested was expressed under the *snb‐1* promoter. To rule out the possibility that *smrd‐1 Δ#1* was affecting the *aex‐3* promoter itself rather than modifying tau phenotypes, we crossed *smrd‐1 Δ#1* with a strain that carries TDP‐43 M337V expressed under *aex‐3* promoter. However, we observed no rescue further confirming the specificity of *smrd‐1* suppression in tauopathy models (Figure S1e). Taken together, these results suggest loss of functional *smrd‐1* specifically suppress neuronal dysfunction in tauopathy models.

### 3.2 *smrd‐1* Deficiency Reduces Tau Protein Expression and Transcript Levels

**FIGURE 2:**
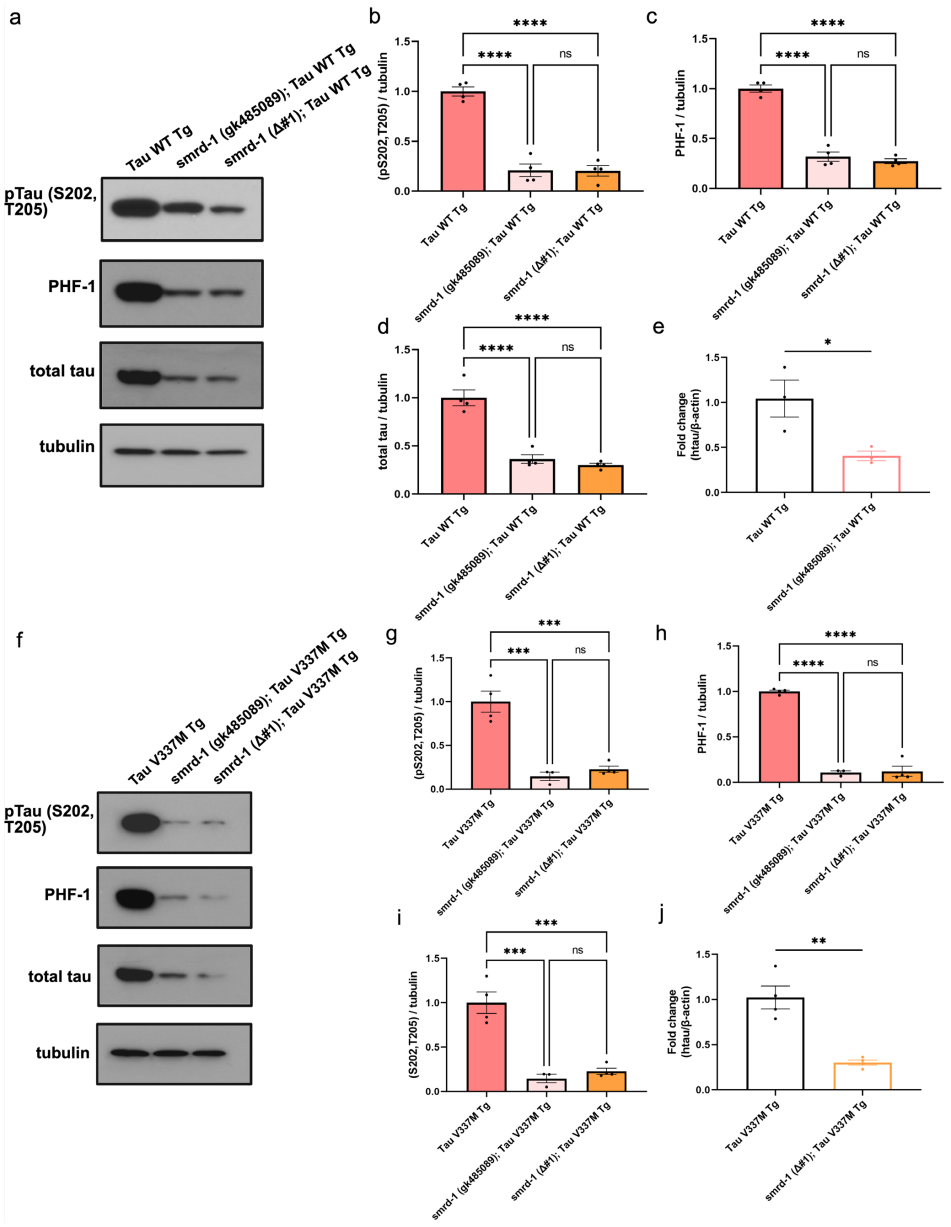
Human tau protein and transcript levels with loss of *smrd‐1* in *C. elegans* models of tauopathy. (a) Immunoblot measuring protein levels of pS202/T205 tau, PHF‐1, total tau and tubulin in day 1 adult lysates of Tau WT Tg animals. (b–d) pTau, PHF‐1, and total tau were significantly reduced in Tau WT Tg animals carrying either*smrd‐1* (*gk484089*) or *smrd‐1* (*Δ#1*). (e) Human Tau mRNA levels were measured by qPCR and showed significant reduction in *smrd‐1* (*Δ#1*); Tau WT animals. (f) Immunoblot measuring protein levels of pS202/T205 tau, PHF‐1, total tau and tubulin in day 1 adult lysates of Tau *V337M* animals. (g–i) pTau, PHF‐1, and total tau were significantly reduced in Tau *V337M* Tg animals carrying either *smrd‐1* (*gk484089*) or *smrd‐1* (*Δ#1*). (j) Human Tau mRNA levels were measured by qPCR and showed significant reduction in *smrd‐1* (*Δ#1*); Tau *V337M* animals. *N* = 3–4 One‐way ANOVA with Tukey's post hoc and Student's *t*‐test. *p < 0.05, **p < 0.01, ***p < 0.001, ****p < 0.0001.

To examine whether *smrd‐1* loss of function impacts tau expression, we assessed protein levels of phosphorylated and total tau in WT tau and *V337M* tau expressing animals with either *smrd‐1* (*gk485089*) *m*utant or *smrd‐1 Δ#1*. In animals expressing WT tau, we observed an 80% reduction in p S202/T205 tau and a 70% reduction in PHF‐1 tau in both *smrd‐1* (*gk485089*) mutant and *smrd‐1 Δ#1*. Interestingly, total tau also diminished by approximately 70% in *smrd‐1* (*gk485089*) mutant and *smrd‐1 Δ#1* (Figure 2a–d). This reduction could result from decreased mRNA transcripts or increased protein turnover. Therefore, we tested *MAPT* transcript levels and found a 60% downregulation of tau mRNA in *smrd‐1* (*gk485089*) mutant (Figure 2e), indicating that the reduction in tau protein is likely due to decreased mRNA levels leading to lower protein translation. In Tau V337M Tg animals, the effects of the loss of *smrd‐1* were also apparent on tau protein and MAPT mRNA levels. pS202/T205 tau was reduced by 80%–85% and PHF‐1 tau showed approximately a 90% reduction. Total tau protein levels were lowered by 75% (Figure 2f–i) and tau transcripts were also downregulated by 70% (Figure 2j). Taken together, the loss of *smrd‐1* either by *smrd‐1 Q747stop* mutation or *smrd‐1 Δ* results in diminished levels of phosphorylated tau and total tau likely driven by decreased *MAPT* transcripts.

### 3.3 *smrd‐1* Deficiency Attenuates Tau‐Induced Neurodegeneration

**FIGURE 3:**
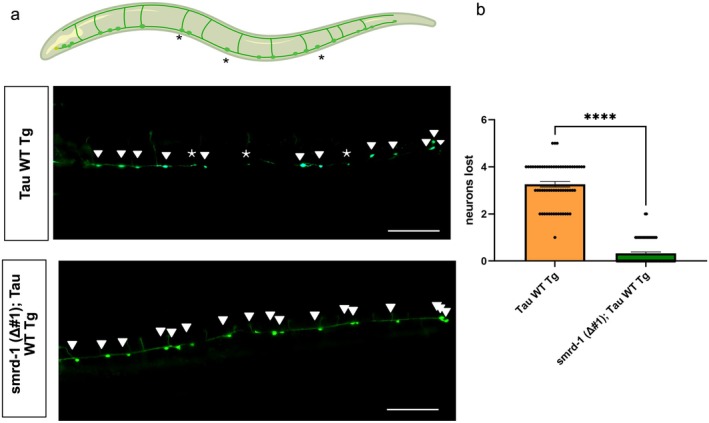
*smrd‐1* deletion rescues tauopathy associated neuronal loss in Tau transgenic animals. (a) Illustration depicts neurons lost in Tau WT Tg animals, which were rescued by *smrd‐1* (*Δ#1*). GABAergic neurons marked by *Punc‐25::GFP* were counted in day 1 adult transgenic animals. Representative images presenting neurons lost and intact neurons in Tau WT Tg animals (upper panel) and *smrd‐1* (*Δ#1*) in Tau WT Tg animals (lower panel). White asterisks indicate lost neurons and filled triangles indicate intact neurons. (b) Quantification of neurons lost indicates significantly fewer neurons were lost in *smrd‐1* (*Δ#1*); Tau WT Tg animals when compared to Tau WT Tg. *N* = 12–15 animals per genotype with four independent repeats. One‐way ANOVA with Tukey's post hoc ****p < 0.0001, additional analyzed data from this set included in Figure S2.

Given that the loss of *smrd‐1* rescues behavioral deficits in tau transgenic animals, we next evaluated effects on tau induced neurodegeneration. Tau WT transgenic animals display progressive age‐dependent neurodegeneration in GABAergic inhibitory motor neurons (Kraemer et al. 2003). To visualize this, we crossed tau WT and *smrd‐1 Δ#1*; tau WT lines with a *Punc‐25::GFP* reporter that fluorescently labels GABAergic motor neurons in vivo. We observed *smrd‐1* loss of function prevented the loss of GABAergic motor neurons in Tau WT Tg (Figure 3a,b). As a control, we confirmed that the number of GABAergic motor neurons in *smrd‐1 Δ#1* was comparable to wild‐type animals (N2) (Figure S2).

### 3.4 Depletion of *SMARCAD1* Lowers Total and Hyperphosphorylated Tau Proteins in Mammalian Cells

**FIGURE 4:**
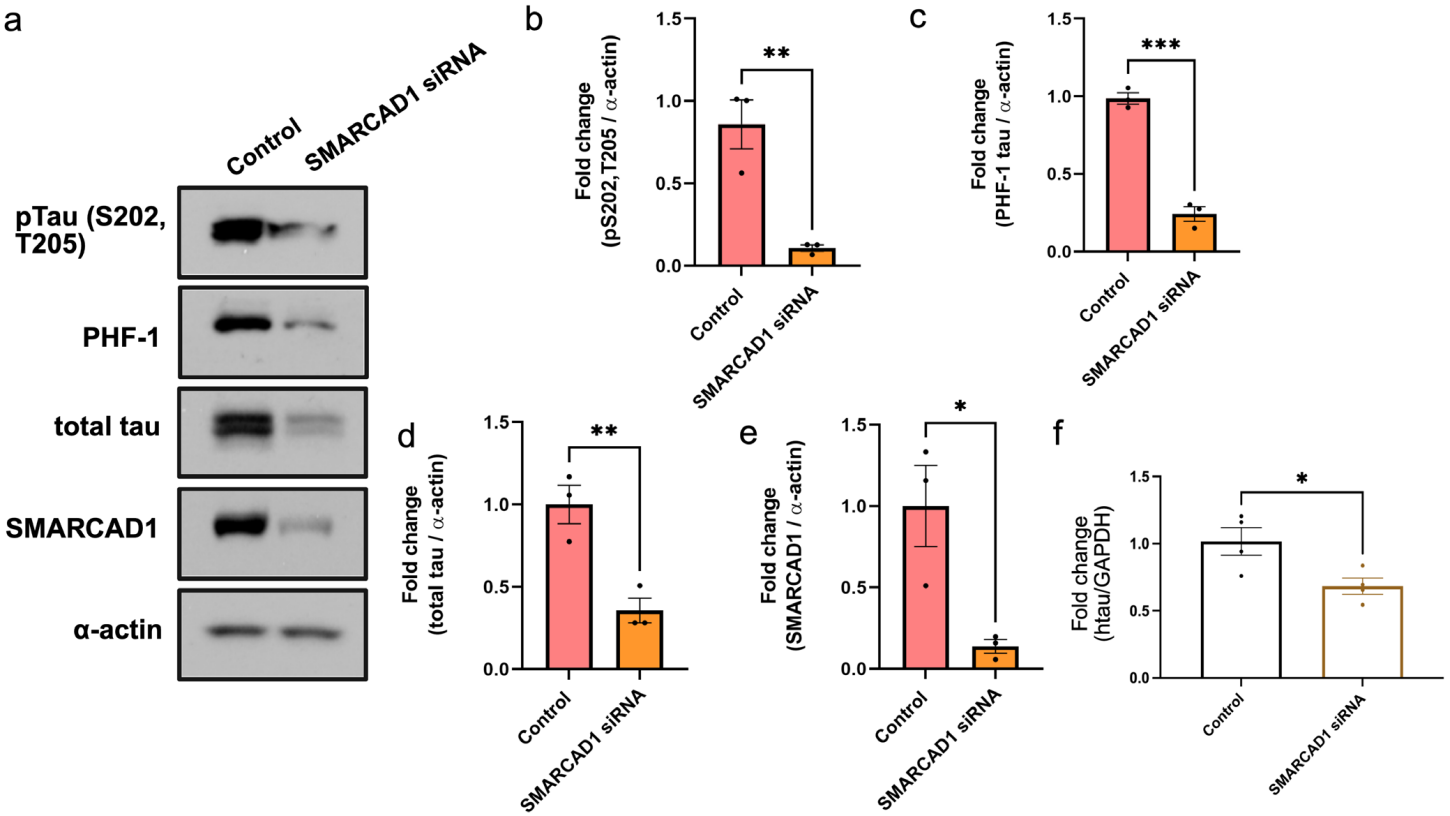
*SMARCAD1* silencing in HEK‐tau cells diminished pathogenic tau protein and mRNA transcripts. (a) Immunoblot measuring of pS202/T205 tau, PHF‐1, total tau, SMARCAD1, and α‐actin in HEK‐tau cells with control treatment and cells treated with *SMARCAD1* siRNA. (b–d) Protein levels of pS202/T205 tau, PHF‐1, and total tau. (e) SMARCAD1 was significantly reduced in *SMARCAD1* siRNA treated HEK‐tau cells. (f) qPCR of human tau showed significant reduction of tau mRNA levels. *N* = 3–4. One‐way ANOVA with Tukey's post hoc and Student's *t*‐test. *p < 0.05, **p < 0.01, ***p < 0.001.

Loss of *smrd‐1* in *C. elegans* models of tauopathy demonstrated a significant rescue of tau mediated behavioral dysfunction and neurodegeneration, and decreased levels of pathological and total tau. We next evaluated whether loss of SMARCAD1 would have a conserved effect on tau in an established mammalian cell model of tauopathy, HEK 293/tau cells. HEK 293/tau stably overexpress human WT (4R1N) tau in a HEK 293 background, resulting in accumulation of abnormal tau species (Guthrie and Kraemer 2011). We used siRNA to target and reduce levels of the mammalian homolog of *smrd‐1*, *SMARCAD1*. Pathological and total tau protein levels in *SMARCAD1* siRNA treated cells were estimated by western blot. siRNA mediated silencing of *SMARCAD1* in HEK 293/tau resulted in 90% reduction in p S202/T205 tau, approximately 75% reduction in PHF‐1 tau and 65% reduction in total tau (Figure 4a–d). Reduced *SMARCAD1* was confirmed by immunoblotting for anti‐SMARCAD1 (Figure 4e). Human MAPT transcript levels were reduced by 35% in *SMARCAD‐1* siRNA treated HEK 293/tau cells (Figure 4f). Similar to attenuation of tau pathology in tau transgenic *C. elegans* models, we observed *SMARCAD1* silencing suppressed tau protein, its phosphorylation and transcripts in mammalian cell model of tauopathy as well, suggesting a conserved mechanism of rescue.

### 3.5 Loss of *smrd‐1* Restores Altered Histone Methylation in Tau Tg Animals

**FIGURE 5:**
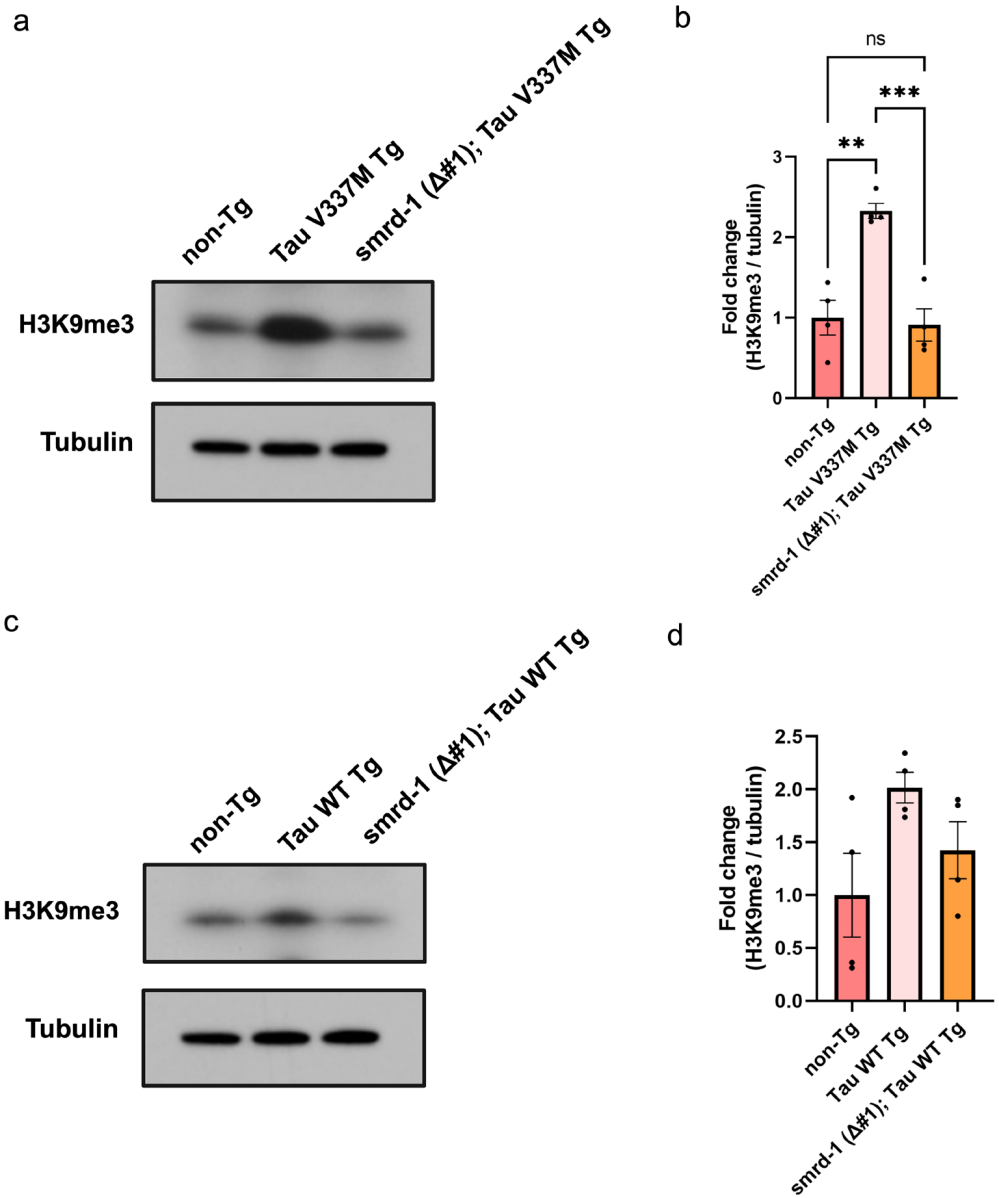
Heterochromatic changes in SMARCAD loss of function. (a) Immunoblot measuring heterochromatin marker H3K9me3 in day 1 adult lysates of animals in Tau *V337M* Tg background. (b) Quantification indicates a significant increase in Tau *V337M* Tg animals as compared to non Tg which is brought back to normal levels in *smrd‐1* (*Δ#1*); Tau *V337M* double transgenic animals. (c) H3K9me3 immunoblot in Tau WT Tg background animals. (d) Quantification indicates a trending but not statistically significant increase in H3K9me3 in Tau WT Tg animals as compared to non Tg animals, and a modest non‐significant decrease in *smrd‐1* (*Δ#1*); Tau WT double transgenic animals. Statistical comparison made with one‐way ANOVA with Tukey's post hoc test, *n* = 3‐4, *p* < 0.05, * *p* < 0.01, ** *p* < 0.001.

**FIGURE 6:**
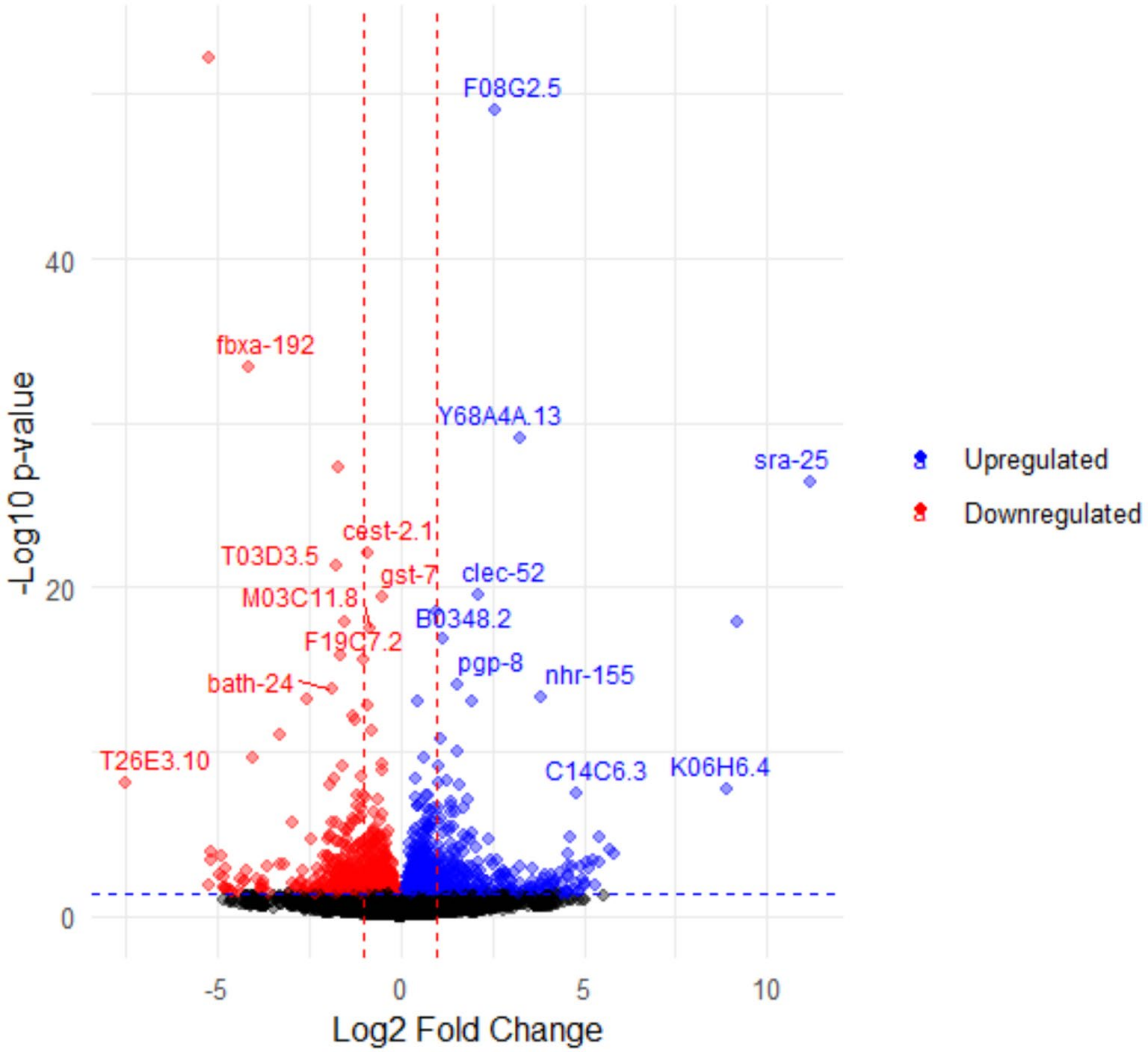
Transcriptomic alterations in *smrd‐1* (*Δ#1*) animals. Volcano plot of bulk RNA sequencing performed on L4 staged *smrd‐1* (*Δ#1*) versus Non Tg animals. Red dots indicated down regulated genes and blue dots indicate upregulated genes.

*SMARCAD1* remodels heterochromatin and is known to be associated with chromatin domains characterized by histone H3 trimethylation at lysine 9, H3K9me3, a hallmark of heterochromatin (Rowbotham et al. 2011; Sachs et al. 2019). To determine whether *smrd‐1* loss of function might impact histone methylation as a surrogate of heterochromatin changes, we examined levels of H3K9me3 in WT and *V337M* tau transgenic day 1 adults by immunoblotting. Surprisingly, we found an approximate 2‐fold increase in the H3K9me3 heterochromatin mark in Tau Tg animals, which was brought back to normal, non‐Tg levels in *smrd‐1 Δ#1;* Tau WT and *smrd‐1 Δ#1;* Tau V337M transgenic animals (Figure 5a–d). This suggests histone methylation increases with tau pathology, and deletion of *smrd‐1* lowers the histone methylation associated with heterochromatin to normal levels in tau transgenic animals. Heterochromatin remodeling can modulate gene expression profiles in various ways and may drive expression of RNAs that can regulate expression of target genes (Lakhotia 2017). To identify gene expression and pathway alterations resulting from *smrd‐1* loss, we performed bulk RNA sequencing on L4 staged *smrd‐1 Δ#1* animals and non‐Tg controls. Overall, 275 differentially expressed genes (*p*‐adj < 0.05) were detected, of which 63 were upregulated and 212 were downregulated transcripts in *smrd‐1 Δ#1* versus non‐Tg (Figure 6, Table S4). The significantly differentially downregulated genes in *smrd‐1 Δ#1* animals included UDP glycosylation, TGF‐β, and transmembrane receptor protein serine/threonine kinase pathway components. Upregulated genes were enriched in UDP‐galactose transmembrane activity, whereas downregulated genes showed enrichment in UDP‐glycosyltransferase activity. Taken together, these data suggest broad changes occur in the transcriptome consistent with chromatin remodeling changes.

### 3.6 SMARCAD1 Lowering Occurs in Alzheimer's Disease Subjects With MSUT2 Depletion

**FIGURE 7:**
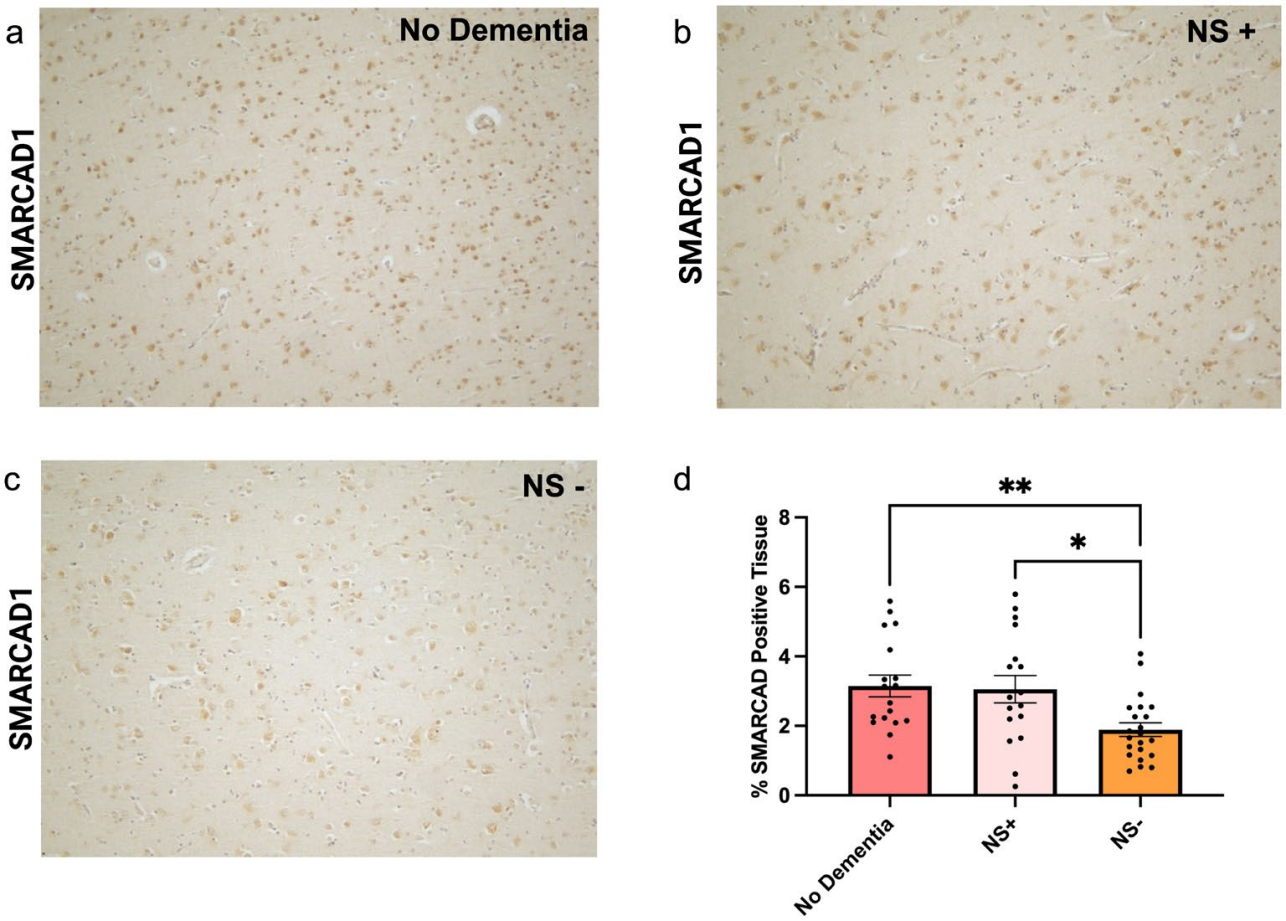
SMARCAD1 immunostaining reduced in postmortem tissue with reduced MSUT2 staining. Medial Temporal gyrus of cognitively normal and dementia subjects were stained for MSUT2 (marker of Nuclear speckle, NS) and SMARCAD1. Subjects were categorized as NS+ and NS− based on MSUT2 staining. (a–c) SMARCAD1 staining in (a) controls, (b) Dementia NS+, and (c) Dementia NS− subjects. (d) Quantification of immunohistochemistry showed percent positive tissue for SMARCAD1 staining in Dementia with NS− subjects as compared to Dementia NS+ and healthy controls. One‐way ANOVA with Bonferroni's post hoc analysis. **p < 0.01, *p < 0.05.

Previously, it has been shown that recessive loss‐of‐function mutations in the *sut‐2* locus suppress the Unc phenotype, tau aggregation and neurodegenerative changes caused by human tau in *C. elegans* (Guthrie et al. 2009). Like *smrd‐1*, *sut‐2* is a neuronal nuclear speckle resident protein involved in tauopathy mechanism in *C. elegans*. Furthermore, H3K9me3 modification marks nuclear speckle adjacent heterochromatin typically associated with active gene expression (Yu et al. 2025). Loss of the *sut‐2* mammalian homolog, *Msut2*, also showed protection against tau pathology and gliosis in a murine PS19 tauopathy model. In human AD subjects, depletion of MSUT2 staining correlated with earlier onset of AD, more severe tau neuropathology, and worsened neurodegeneration, suggesting MSUT2 abundance controls tauopathy severity. (Wheeler et al. 2019). To examine whether a similar depletion of SMARCAD1 occurs in disease, we analyzed SMARCAD1 staining in AD subjects but did not find any significant changes as compared to no dementia controls. However, when we segregated AD subjects based on MSUT2 staining as MSUT2 present (MSUT2+) and MSUT2 depleted (MSUT2−/severe tauopathy) subjects. We observed SMARCD1 staining was significantly lower in MSUT2‐ subjects as compared to MSUT2+ and no dementia subjects (Figure 7a–d) suggesting a parallel relationship between SMARCAD1 and tauopathy.

## 4 Discussion

Using forward genetic screening, we identified two putative loss of function mutations in *smrd‐1* that suppressed tauopathy associated neuronal dysfunction in tau transgenic *C. elegans* . To confirm the suppression, we obtained an additional putative loss of function *smrd‐1* allele and generated two *smrd‐1* deletion alleles using the CRISPR‐Cas9 genome editing system (Kow et al. 2021). All *smrd‐1* alleles rescued the tauopathy associated behavioral phenotype and reduced neurodegeneration. Loss of *smrd‐1* also reduced total and phosphorylated tau protein levels in both Tau WT and Tau *V337M* animals, likely through decreased tau mRNA. Heterozygous expression of *smrd‐1* showed modest yet significant rescue of the behavioral phenotype in Tau WT animals, making it a promising target. *Smrd‐1* mediated rescue seems to be specific to tauopathy, as loss of *smrd‐1* in TDP‐43 expressing animals had no change in TDP‐43 associated neuronal dysfunction.

To understand whether loss of SMARCAD1 can rescue tauopathy in mammalian cells, we silenced SMARCAD1 in HEK‐tau cells and observed a similar reduction in phosphorylated and total tau protein levels and human tau transcripts. This suggests SMARCAD1 has a conserved mechanism suppressing tauopathy as it rescues pathogenic tau across different model systems.

SMARCAD1 is a DNA helicase with ATP‐dependent nucleosome remodeling capabilities and is required for maintenance of heterochromatin organization. While SMARCAD1 regulates global H3K9me3 levels on chromatin (Rowbotham et al. 2011), there are also reports of static global H3K9me3 with regulation of H3K9me3 only at SMARCAD1‐binding sites (Sebastian‐Perez et al. 2025; Xiao et al. 2017). We assessed histone methylation associated with heterochromatin status by measuring the repressive marks, H3K9me3 levels, in the presence and absence of *smrd‐1* in *C. elegans* . Interestingly, tau Tg animals expressed higher H3K9me3 as compared to non‐Tg animals. This elevated H3K9me3 was brought back to normal levels in *smrd‐1 Δ#1;* tau Tg, confirming loss of *smrd‐1* lowers heterochromatin levels. The restoration of heterochromatin to wild‐type levels does not necessarily indicate its precise re‐establishment at the original genomic regions and the effect of *smrd‐1* on re‐establishment needs further investigation. It has been shown that AD subjects have elevated H3K9me3 immunoreactivity compared to controls, and they also exhibit differences in locations of H3K9me3 marks. In these subjects, H3K9me3‐enriched genes and chromatin regions are associated with synaptic transmission, neuronal differentiation, and cell motility, which includes *BDNF*, *GABBR1*, *GABBR2*, and *GPRASP1* (Lee et al. 2020). Increased H3K9me3 in our tau transgenic animals could indicate increased repression of these synaptic genes. Although loss of *smrd‐1* restores gross H3K9me3 levels in tau transgenic animals, further studies are needed to determine whether *smrd‐1 Δ#1;* tau Tg animals have similar marked H3K9me3 genomic regions as compared to non‐Tg animals. While we propose chromatin remodeling alters tauopathy, tau has also been shown to affect chromatin compaction (Rico et al. 2021). Since we observed elevated H3K9me3 in tau‐transgenic animals, we propose that tau regulates heterochromatin through *smrd‐1*. However, we also observe clearance of MAPT mRNA and protein upon *smrd‐1* deletion. This suggests a possible feedback loop mechanism of regulation worth further investigation. Tau‐transgenic *Drosophila* and mice were shown to have widespread heterochromatin loss and restoring heterochromatin suppressed tau‐mediated neuronal apoptosis in *Drosophila*, similar to our observation in *C. elegans* (Frost et al. 2014). We suggest SMARCAD1 regulates tau mRNA expression while increased tau levels could in turn alter heterochromatin through SMARCAD1 in a mechanism conserved in both *C. elegans* and *Drosophila*. Our data in hand does not directly address this mechanism, but further genomic investigations are warranted to confirm the heterochromatin mechanism of tauopathy also occurs in mammalian systems.

Transcriptomic analysis of *smrd‐1 Δ#1* animals revealed alteration in several pathways including UDP glycosylation, TGF‐β, and transmembrane receptor protein serine/threonine kinase. Upregulated genes were enriched in UDP‐galactose transmembrane activity whereas downregulated genes showed enrichment in UDP‐glycosyltransferase activity. This indicates overall reduction in UDP glycosylation. While pathological tau is shown to be glycosylated in AD (Liu et al. 2002; Losev et al. 2019), this reduction in UDP glycosylation might contribute to the reduction in hyperphosphorylated tau accumulation in *smrd‐1 Δ#1* animals. Downregulated genes also showed enrichment in biological processes that indicated increases in TGF‐β pathway and transmembrane receptor protein serine/threonine kinase. TGF‐β has neuroprotective functions in both nematodes (Baltaci et al. 2022) and in humans with AD (Caraci et al. 2009). Increases in TGF‐β pathway could be one of the mechanisms how *smrd‐1 Δ#1* animals rescue tauopathy mediated neurodegeneration. While these pathways shed light on *smrd‐1* mechanisms that may contribute to reduced hyperphosphorylated tau protein levels and neurodegeneration, mechanisms controlling the reduction in tau transcripts require additional study.

As mentioned above, SMARCAD1 and MSUT2 are co‐localized in nuclear speckles. SMARCAD1 controls HeK9me3 methylation which demarcates heterochromatin proximity to nuclear speckles; HeK9me3 is associated with actively expressing genes (Yu et al. 2025). Furthermore, disruption of the nuclear speckle scaffold protein SRRM2 disrupts speckles and heterochromatin associated gene expression (Hu et al. 2019). In severe AD cases, we know the SRRM2 co‐localizing nuclear speckle protein MSUT2 becomes depleted as compared to normal controls. It has been hypothesized that in presence of severe tauopathy, MSUT2+ neurons degenerate leaving behind MSUT2‐ neurons that survive tauopathy (Wheeler et al. 2019). In these same severe AD cases SMARCAD1 protein appears to decrease while SMARCAD1 remained relatively unchanged in MSUT2+ subjects. While the interpretation of the SMARCAD1 protein levels and its contribution to the pathogenesis based on staining in autopsy brains is difficult, we suggest decreased SMARCAD1 may represent a compensatory mechanism to cope with enhancing tau pathology in MSUT2 depleted cases.

## 5 Conclusion

In this study, we report a novel tauopathy suppressor that ameliorates accumulation of toxic tau species and neurodegeneration in *C. elegans* tau transgenic models and in mammalian cells overexpressing human tau. Based on assessment of the heterochromatin levels, we propose SMARCAD1‐mediated tauopathy rescue involves epigenetic alterations. Further studies may pinpoint exact pathways that are altered downstream of the heterochromatin changes that contribute to the tauopathy rescue, which in turn may highlight new therapeutic ideas for tauopathy.

## Author Contributions

V.S.J., R.L.K., and B.C.K. designed research; V.S.J., A.D.B., M.B., and P.J.M. performed experiments; C.S.L. provided human post‐mortem brain specimens, neuropathological analyses, and disease staging; V.S.J., R.L.K., P.J.M., N.F.L., and B.C.K. analyzed, interpreted data, and wrote the paper; V.S.J., A.D.B., M.B., P.J.M., C.S.L., R.L.K., N.F.L., and B.C.K. edited the paper.

## Funding

This work was supported by grants from the United States (U.S.) Department of Veterans Affairs [I01BX005762 to N.F.L. and IK6BX006467 to B.C.K.] and National Institutes of Health [R01AG066729 to N.F.L., R01NS0064131 to B.C.K., P30AG066509 and U19AG066567 to C.S.L.].

## Ethics Statement

Informed consent for research brain donation was obtained according to protocols approved by the UW and KPWHRI Institutional Review Boards.

## Consent

The authors have nothing to report.

## Conflicts of Interest

The authors declare no conflicts of interest.

## Supporting information


**Figure S1:** (a) *smrd‐1* (*bk2196*) vs. Non Tg showed no significant changes in swimming assay. (b) Non Tg, *smrd‐1* (*gk485089*), *smrd‐1* (*Δ#1*), *smrd‐1* (*Δ#2*) behaved similarly with no significant changes in swimming assay. (c) Hemizygous *smrd‐1* (*bk2935*) rescues tau associated motor deficits in Tau WT Tg animals in comparison to Non Tg animals. (d, e) *smrd‐1* (*Δ#1*) does not rescue TDP‐43 associated motor deficits in TDP‐43 M337V Tg animals when expressed under (e) *snb‐1* promoter or (f) *aex‐3* promoter. Data presented as mean ± SEM frequency of body bends (turns/min) measured over the course of a minute of swimming in day 1 adults and analyzed by One‐way ANOVA with Tukey's post hoc test.
**Figure S2:** Quantification of neurons lost in animals expressing GFP in GABAergic neurons with WT *smrd‐1* and with *smrd‐1* (*Δ#1*). *N* = 12–15 animals per genotype with four independent repeats. One‐way ANOVA with Tukey's post hoc.
**Figure S3:** Bubble graph of gene enrichment for DAVID GO terms Biological processes (BP), Cellular Compartment (CC) and Molecular functions (MF) in significantly (a) upregulated genes and (b) downregulated genes. All the significant processes *p* < 0.05 are plotted.
**Table S1:** *C. elegans* strain details.
**Table S2:** Oligonucleotide sequences for qRT‐PCR primers and siRNAs.
**Table S3:** Demographic and clinical characteristics of human subjects for SMARCAD1 immunohistochemistry.
**Table S4:** Differential gene expression for smrd‐1 deletion vs. non‐Tg.

## Data Availability

Analyzed data are included within the manuscript and in Supporting Information files. RNA sequencing data are available on the GEO website GSE297216.
