# Supplementary material for: Loss of SMARCAD1 Mitigates Tauopathy
Source: Aging Cell. 2026 May 27;25(6):e70543. doi: 10.1111/acel.70543 (PMC13240146; doi:10.1111/acel.70543)
Supplement: Supplementary file 1 — Figure S1: (a) smrd‐1 (bk2196) vs. Non Tg showed no significant changes in swimming assay. (b) Non Tg, smrd‐1 (gk485089), smrd‐1 (Δ#1), smrd‐1 (Δ#2) behaved similarly with no significant changes in swimming assay. (c) Hemizygous smrd‐1 (bk2935) rescues tau associated motor deficits in Tau WT Tg animals in comparison to Non Tg animals. (d, e) smrd‐1 (Δ#1) does not rescue TDP‐43 associated motor deficits in TDP‐43 M337V Tg animals when expressed under (e) snb‐1 promoter or (f) aex‐3 promoter. Data presented as mean ± SEM frequency of body bends (turns/min) measured over the course of a minute of swimming in day 1 adults and analyzed by One‐way ANOVA with Tukey's post hoc test. Figure S2: Quantification of neurons lost in animals expressing GFP in GABAergic neurons with WT smrd‐1 and with smrd‐1 (Δ#1). N = 12–15 animals per genotype with four independent repeats. One‐way ANOVA with Tukey's post hoc. Figure S3: Bubble graph of gene enrichment for DAVID GO terms Biological processes (BP), Cellular Compartment (CC) and Molecular functions (MF) in significantly (a) upregulated genes and (b) downregulated genes. All the significant processes p < 0.05 are plotted. Table S1: C. elegans strain details. Table S2: Oligonucleotide sequences for qRT‐PCR primers and siRNAs. Table S3: Demographic and clinical characteristics of human subjects for SMARCAD1 immunohistochemistry. Table S4: Differential gene expression for smrd‐1 deletion vs. non‐Tg. [file ACEL-25-e70543-s001.zip › acel70543-sup-0001-TableS2-FigureS1-S3@Supplementary_information_r_11_7_2025.pdf]

Supplementary Information

Table S2

**Primers/siRNA sequence**	**Sequence**
human *MAPT* for Tau Tg worms	GTGTGGCTCATTAGGCAACATCC
	CGTTCTCGCGGAAGGTCAG
rpl-32	GGTCGTCAAGAAGAAGCTCACCAA
	TCTGCGGACACGGTTATCAATTCC
act-1	AGCCATCCTTCTTGGGTATG
	ATTCCTGGGTACATGGTGGT
Human *MAPT* for HEK-tau cells	GTGTGGCTCATTAGGCAACATCC
	CGTTCTCGCGGAAGGTCAG
GAPDH	CTGGGCTACACTGAGCACCAG
	CCAGCGTCAAAGGTGGAG
SMARCAD1 siRNA 13.6	5 ' - UCUGAAAGGAUUCAUCUAAUUGATG - 3’<br>3 ' - AAAGACUUUCCUAAGUAGAUUAACUAC - 5 '

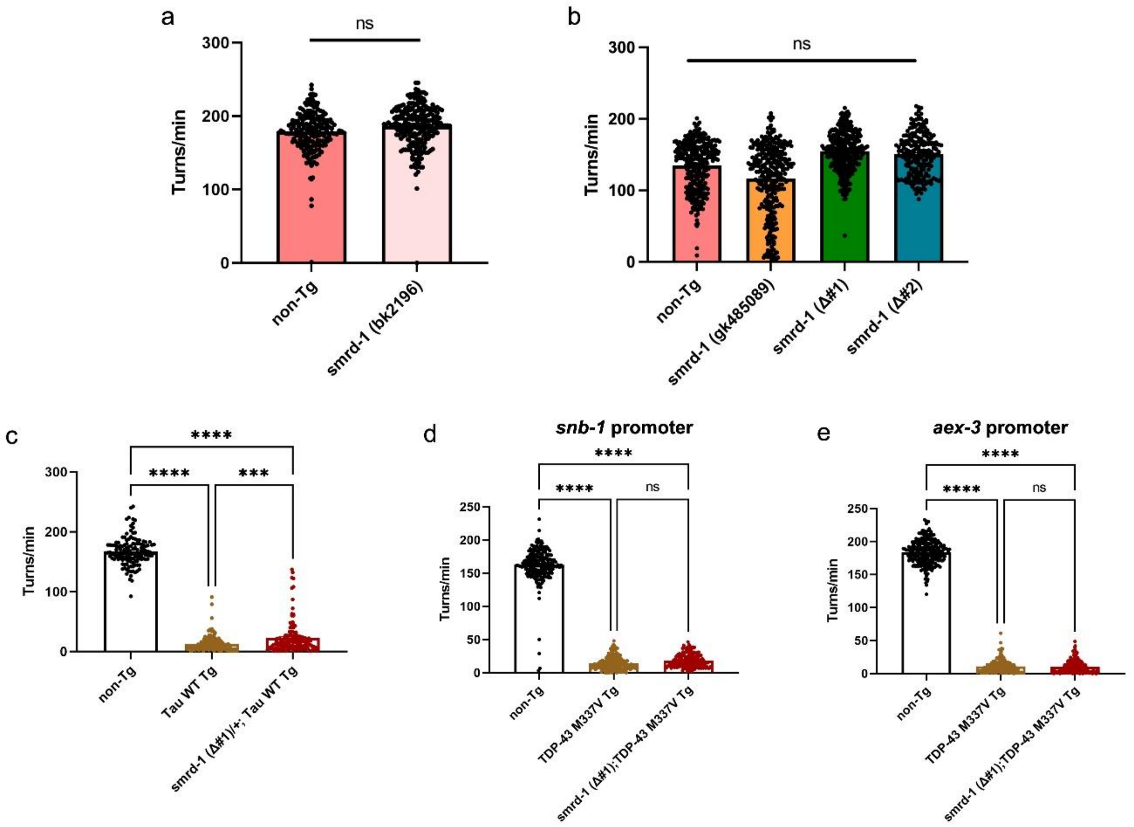

Figure S1

a. *smrd-1(bk2196)* vs Non Tg showed no significant changes in swimming assay. b. Non Tg, *smrd-1(gk485089), smrd-1 (Δ#1), smrd-1 (Δ#2)* behaved similarly with no significant changes in swimming assay. c. Hemizygous *smrd-1 (bk2935)* rescues tau associated motor deficits in Tau WT Tg animals in comparison to Non Tg animals. d-e. *smrd-1 (Δ#1)* does not rescue TDP-43 associated motor deficits in TDP-43 M337V Tg animals when expressed under e. *snb-1* promoter or f. *aex-3* promoter. Data presented as mean ± SEM frequency of body bends (turns/min) measured over the course of a minute of swimming in day 1 adults and analyzed by One way ANOVA with Tukey's post hoc test.

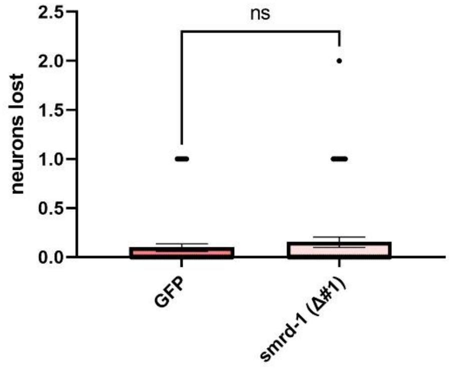

Fig.S2

Quantification of neurons lost in animals expressing GFP in GABAergic neurons with WT *smrd-1* and with *smrd-1(Δ#1)*. N=12-15 animals per genotype with 4 independent repeats. One-way ANOVA with Tukey’s post hoc.

a

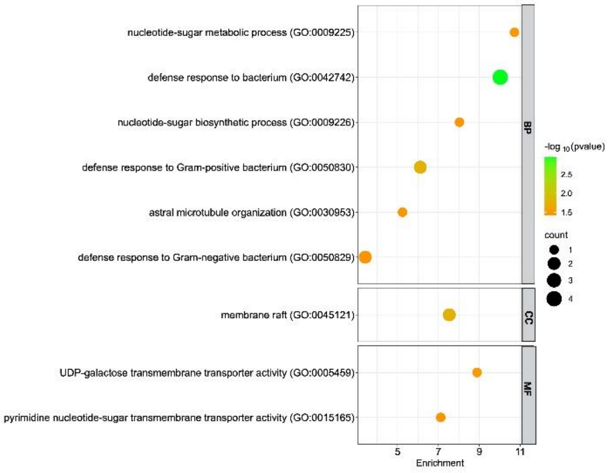

b

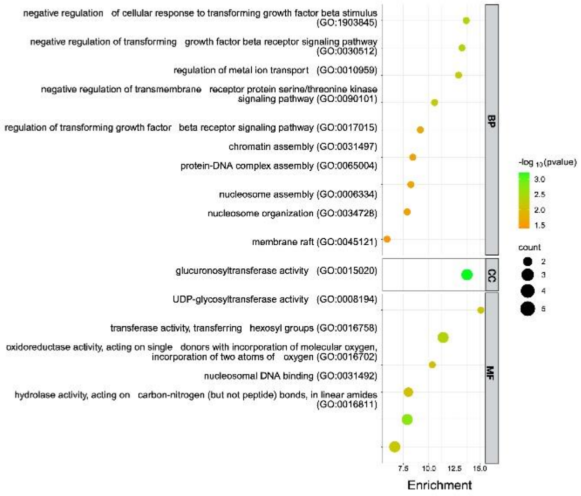

Figure S3

Bubble graph of gene enrichment for DAVID GO terms Biological processes (BP), Cellular Compartment (CC) and Molecular functions (MF) in significantly a. upregulated genes and b. downregulated genes. All the significant processes P<0.05 are plotted.
